# Population Pharmacokinetics of Unbound Ceftolozane and Tazobactam in Critically Ill Patients without Renal Dysfunction

**DOI:** 10.1128/AAC.01265-19

**Published:** 2019-09-23

**Authors:** Fekade B. Sime, Melissa Lassig-Smith, Therese Starr, Janine Stuart, Saurabh Pandey, Suzanne L. Parker, Steven C. Wallis, Jeffrey Lipman, Jason A. Roberts

**Affiliations:** a University of Queensland Centre for Clinical Research, Faculty of Medicine, The University of Queensland, Brisbane, Australia; b School of Pharmacy, Centre for Translational Anti-infective Pharmacodynamics, The University of Queensland, Brisbane, Australia; c Department of Pharmacy and Intensive Care Medicine, Royal Brisbane and Women’s Hospital, Brisbane, Australia; d Division of Anaesthesiology Critical Care Emergency and Pain Medicine, Nîmes University Hospital, University of Montpellier, Nîmes, France

**Keywords:** ceftolozane, critically ill, dosing, intensive care unit, population pharmacokinetics, tazobactam

## Abstract

Evaluation of dosing regimens for critically ill patients requires pharmacokinetic data in this population. This prospective observational study aimed to describe the population pharmacokinetics of unbound ceftolozane and tazobactam in critically ill patients without renal impairment and to assess the adequacy of recommended dosing regimens for treatment of systemic infections. Patients received 1.5 or 3.0 g ceftolozane-tazobactam according to clinician recommendation. Unbound ceftolozane and tazobactam plasma concentrations were assayed, and data were analyzed with Pmetrics with subsequent Monte Carlo simulations. A two-compartment model adequately described the data from twelve patients. Urinary creatinine clearance (CL_CR_) and body weight described between-patient variability in clearance and central volume of distribution (*V*), respectively. Mean ± standard deviation (SD) parameter estimates for unbound ceftolozane and tazobactam, respectively, were CL of 7.2 ± 3.2 and 25.4 ± 9.4 liters/h, *V* of 20.4 ± 3.7 and 32.4 ± 10 liters, rate constant for distribution of unbound ceftolozane or tazobactam from central to peripheral compartment (Kcp) of 0.46 ± 0.74 and 2.96 ± 8.6 h^−1^, and rate constant for distribution of unbound ceftolozane or tazobactam from peripheral to central compartment (Kpc) of 0.39 ± 0.37 and 26.5 ± 8.4 h^−1^. With dosing at 1.5 g and 3.0 g every 8 h (q8h), the fractional target attainment (FTA) against Pseudomonas aeruginosa was ≥85% for directed therapy (MIC ≤ 4 mg/liter). However, for empirical coverage (MIC up to 64 mg/liter), the FTA was 84% with the 1.5-g q8h regimen when creatinine clearance is 180 ml/min/1.73 m^2^, whereas the 3.0-g q8h regimen consistently achieved an FTA of ≥85%. For a target of 40% of time the free drug concentration is above the MIC (40% *f*T_>MIC_), 3g q8h by intermittent infusion is suggested unless a highly susceptible pathogen is present, in which case 1.5-g dosing could be used. If a higher target of 100% *f*T_>MIC_ is required, a 1.5-g loading dose plus a 4.5-g continuous infusion may be adequate.

## Abstract (precis)

Evaluation of dosing regimens for critically ill patients requires pharmacokinetic data in this population. This prospective observational study aimed to describe the population pharmacokinetics of unbound ceftolozane and tazobactam in critically ill patients without renal impairment and to assess the adequacy of recommended dosing regimens for treatment of systemic infections. Patients received 1.5 or 3.0 g ceftolozane-tazobactam according to clinician recommendation.

## TEXT

Ceftolozane-tazobactam is the most active beta-lactam antibiotic against Pseudomonas aeruginosa that is currently available in the market (1). Susceptibility surveillance programs report that the majority of P. aeruginosa clinical isolates (97.5%) remain susceptible (2). The current approved indications are treatment of complicated intra-abdominal infections (cIAI), complicated urinary tract infections (cUTI) (3), and, more recently, hospital-acquired bacterial pneumonia and ventilator-associated bacterial pneumonia. Since approval in 2014, its use has not be restricted to these indications, with some documented off-label uses against multidrug-resistant (MDR) *Pseudomonas* infections, including septicemia/bacteremia (4–6) and possibly extending to other relatively rare infections, including meningitis/ventriculitis. Indeed, some expert opinion has suggested that the place in therapy could encompass all infections susceptible to this agent that are caused by MDR *Pseudomonas* and other extended-spectrum-beta-lactamase-producing Gram-negative bacilli, where it could be considered as a carbapenem-sparing alternative (7). Although most of the off-label use case reports demonstrate successful ceftolozane-tazobactam therapy against multidrug-resistant strains of P. aeruginosa, including those with carbapenem resistance, unfortunately, some of the reports also highlight a potential risk of emergence of resistance during treatment. For example, in the treatment of MDR P. aeruginosa pneumonia, Katchanov et al. (4) reported the emergence of very high resistance to ceftolozane-tazobactam during the course of therapy. Escolà-Vergé et al. (6) also reported development of resistance during therapy with both the low-dose (1.5 g every 8 h [q8h] for urinary tract and soft tissue infections) and high-dose (3.0 g every 8 h for respiratory infections) regimens, with an increase in MIC ranging from 8-fold to >85-fold.

The development of resistance during treatment is likely to be multifactorial. In intensive care unit (ICU) patients, subtherapeutic exposure from standard doses of antibiotics is one of the major contributing factors to emergence of resistance (8). Numerous clinical studies have reported subtherapeutic antibiotic concentrations in ICU patients across different antibiotic classes while using standard dosing regimens (9, 10). This is related to marked changes in the pharmacokinetics (PK) of antibiotics in the critically ill arising from disease-related physiological changes, primarily due to an intense systemic inflammatory response syndrome (SIRS) that is triggered by infectious or noninfectious insults such as sepsis, septic shock, burns, and trauma (10–12). During the progression of SIRS, numerous endogenous inflammatory mediators can cause a hyperdynamic state characterized by high cardiac output, increased renal blood flow, and glomerular hyperfiltration, which ultimately increase clearance (CL) of renally cleared antibiotics (13). In addition, SIRS can cause a capillary leak syndrome and consequent fluid shift into interstitial space, which in turn increases the volume of distribution of hydrophilic antibiotics and thereby decrease plasma/tissue concentrations (14). In patients with hypalbuminaemia, reduced plasma-oncotic pressure further augments fluid shifts, leading to increases in volume of distribution for some drugs. Hypoalbuminemia also results in a substantial increase in the unbound plasma concentration, particularly for highly protein-bound antibiotics, which means that more drug distributes into the interstitial space, with the increased fluid shift thereby accelerating the expansion in volume of distribution (15). However, although the influence of hypoalbuminemia has been described for highly protein-bound drugs, is it less frequently reported with drugs that are protein bound at low levels. Nevertheless, regardless of their protein binding, the PK of hydrophilic antibiotics, such as the beta-lactams, that normally distribute into the extracellular water and undergo predominantly renal elimination often change because of critical illness (11).

The clinical formulation of ceftolozane-tazobactam (Zerbaxa) comprises the combination of ceftolozane sulfate (molecular weight of 764.77) and tazobactam sodium (molecular weight of 322.28) in a 2:1 ratio, both of which are freely soluble in water (16). Owing to these physicochemical properties, the distribution of ceftolozane and tazobactam is generally limited to extracellular water, and their elimination is predominantly via renal excretion (17). These properties make ceftolozane-tazobactam vulnerable to disease-related PK alterations in the critically ill (11). It is now well established that designs of dosing regimens for use in the critically ill population that are based on dose finding/PK studies in healthy volunteers and/or noncritically ill patient populations do not always result in optimal regimens for use in ICU patients (10). It is therefore very important to assess dose recommendations for new agents like ceftolozane-tazobactam based on clinical PK data in this specific patient population.

The aim of this study was, therefore, to describe the population PK of unbound ceftolozane and tazobactam in critically ill patients without renal impairment and to assess the adequacy of recommended dosing regimens.

## RESULTS

Patient demographics and clinical data are summarized in Table 1. From twelve critically ill patients, 133 unbound concentration-time data points were available for population pharmacokinetic analysis.

**TABLE 1:** Characteristics of study participants^*a*^

Characteristic	*n* (%) or median (IQR)
Age (yr)	56 (52–61)
Sex	
Male	4 (33)
Female	8 (67)
Body mass index (kg/m^2^)	28.5 (22.1–32.9)
Wt (kg)	79.5 (64–99)
Serum creatinine (μmol/liter)	46 (39–77)
Urinary creatinine clearance (ml/min/1.73 m^2^)	107 (74–145)
Albumin (g/liter)	25 (19–28)
Alanine transaminase (IU/ml)	35 (23–45)
Aspartate transaminase (IU/ml)	37 (30–67)
Alkaline phosphatase (IU/ml)	102 (75–222)
Total bilirubin (μmol/liter)	12 (7–26)
APACHE II score (on admission)	19.5 (16–26)
SOFA score	6 (3–8)
Site/source of infection	
Blood	2 (17)
CNS abscess	3 (23)
Intra-abdominal	3 (27)
Lung	9 (75)
Urinary tract	1 (8)
Vascular access	1 (8)
Patients with positive culture	12 (100)
Organism isolated	
Acinetobacter baumannii complex	1 (8)
Aspergillus flavus complex	1 (8)
Candida albicans	3 (27)
Candida glabrata complex	1 (8)
Citrobacter koseri	1 (8)
Enterobacter cloacae	2 (17)
Enterococcus faecium	1 (8)
Escherichia coli	3 (27)
Haemophilus influenzae	1 (8)
Klebsiella pneumoniae	1 (8)
Proteus mirabilis	1 (8)
Pseudomonas aeruginosa	2 (17)
Staphylococci	1 (8)
Staphylococcus epidermis	1 (8)
Streptococcus salivarius	1 (8%)

a Abbreviations: IQR, interquartile range; APACHE II, acute physiology and chronic health evaluation II; SOFA, sequential organ failure assessment; CNS, central nervous system.

**FIG 1:**
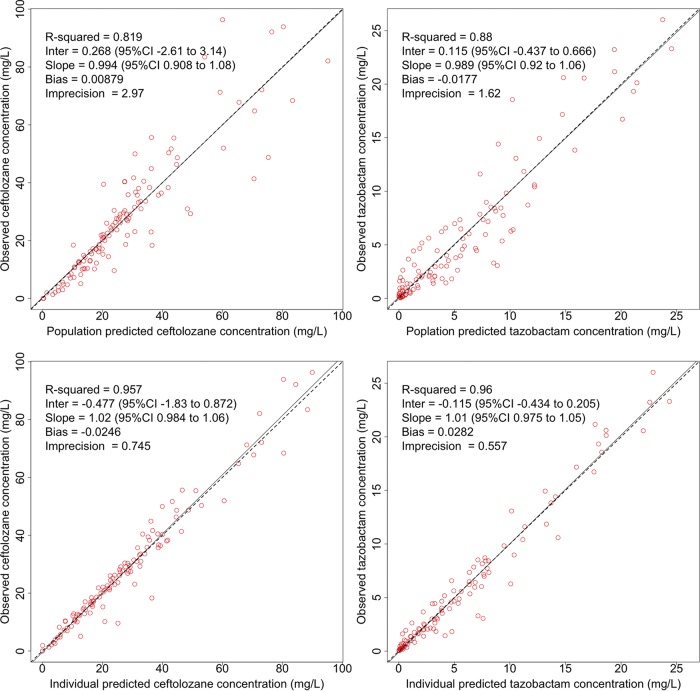
Observed versus predicted goodness-of-fit plots for unbound ceftolozane and tazobactam concentrations. Top panel, population predicted concentrations; bottom panel, individual predicted concentrations.

**FIG 2:**
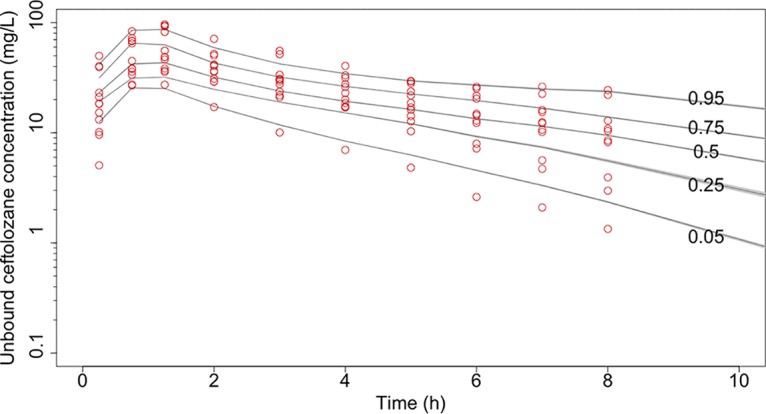
Visual predictive check plot for unbound ceftolozane concentrations. Circles, observed concentrations; lines, simulated concentrations at the designated quantile given by the number on the line.

A two-compartment structural model with linear elimination resulted in the lowest objective function values and best goodness-of-fit plots (log-likelihood ratio [LLR] of 723) compared to those of a one-compartment structural model (LLR of 795). Covariate analysis showed that ceftolozane and tazobactam clearance linearly increased with an increase in urinary creatinine clearance (CL_CR_). The final covariate model for clearance of both ceftolozane and tazobactam was expressed as CL = intercept + slope · CL_CRurinary_, where CL_CRurinary_ is measured urinary creatinine clearance. Total body weight (WT) was related to volume of distribution of the central compartment (*V*_1_) for both ceftolozane (*V*_1_ = *V* · WT/80) and tazobactam (*V*_1_ = *V* · [WT/80]^0.75^), where *V* is the typical value of the central volume of distribution. The introduction these covariates into the structural model substantially reduced the LLR to 711. Parameter estimates for the final models are given in Table 2. The individual and population predicted versus observed unbound concentration plots for ceftolozane and tazobactam are given in Fig. 1. A visual predictive check plot based on 1,000 simulations with the final model is given in Fig. 2.

**TABLE 2:** Pharmacokinetic parameter estimates for the final covariate model^*a*^

Drug and parameter	Mean	SD	CV (%)
Ceftolozane			
Intercept	0.86	0.69	80
Slope	6	3.3	54
*V* (liters)	20.4	3.7	18
Kcp (h^−1^)	0.46	0.74	159
Kpc (h^−1^)	0.39	0.37	94
CL (liters/h)^*b*^	7.2	3.2	45
Tazobactam			
Intercept	6.9	5.6	81
Slope	17.5	6.9	40
*V* (liters)	32.4	10	31
Kcp (h^−1^)	2.96	8.69	293
Kpc (h^−1^)	26.5	8.4	32
CL (liters/h)^*a*^	25.4	9.4	37

a Abbreviations: CV, coefficient of variance; *V*, typical volume of distribution of the central compartment; Kcp, rate constant for distribution of unbound ceftolozane or tazobactam from central to peripheral compartment; Kpc, rate constant for distribution of unbound ceftolozane or tazobactam from peripheral to central compartment; CL, clearance.

b Value calculated for the study population.

The probability of target attainment (PTA) for ceftolozane, considering the median urinary creatinine clearance (108 ml/min/1.73 m^2^) and body weight (80 kg) of the study population, for different dosing regimens during the first 24 h and at steady state from 48 to 72 h is given in Table 3 by MIC for different targets (40, 60, and 100% time the free drug concentration is above the MIC [*f*T_>MIC_]). Generally, intermittent dosing regimens of ceftolozane-tazobactam (1.5 g q8h and 3.0 q8h) were adequate to achieve 100% PTA, well above the highest anticipated clinical breakpoint of susceptibility (4 mg/liter for P. aeruginosa), for 40% and 60% *f*T_>MIC_ targets. For the 100% *f*T_>MIC_ target, the 1.5-g q8h intermittent regimens achieved a ≥90% PTA for an MIC of ≤2 mg/liter and the 3.0-g q8h regimens achieved a ≥90% PTA up to the P. aeruginosa clinical breakpoint (MIC ≤ 4 mg/liter). Loading dose (LD) plus continuous infusion (CI) regimens (1.5-g LD plus 4.5-g CI and 3.0-g LD plus 9-g CI) were able to provide optimal exposure (≥90% PTA) up to MICs of 8 mg/liter and 16 mg/liter, respectively. The regimen of a 3.0-g LD plus 9.0-g CI in particular achieved high steady-state ceftolozane concentrations of 22.4 (±6.7) and 38 (±11) mg/liter for augmented (180 ml/min/1.73-m^2^) and normal (100 ml/min/1.73-m^2^) creatinine clearance values, respectively. On the other hand, for tazobactam, all simulated dosing regimens had a 100% probability of achieving the recommended target of 20% *f*T_>1mg/liter_. Table 4 present the FTA for ceftolozane against the P. aeruginosa EUCAST MIC distribution, considering steady-state exposure, for increasing values of urinary creatinine clearance. For directed therapy, i.e., for isolates with MICs within the susceptibility range, the 1.5-g q8h intermittent regimen achieved the optimal FTA (>85%) even in patients with urinary creatinine clearance as high as 180 ml/min/1.73 m^2^, except when targeting a 100% *f*T_>MIC_, whereas the 3.0-g q8h dosing regimen achieve the optimal FTA for all targets and high creatinine clearance values for directed therapy. On the other hand, for empirical coverage against the entire MIC distribution, the 1.5-g q8h regimen appears to be suboptimal in patients with high creatinine clearance (>140 ml/min/1.73 m^2^) when considering the standard target of 40% *f*T_>MIC_ and even in patients with creatinine clearance as low as 100 ml/min/1.73 m^2^ if high PK/pharmacodynamic (PD) targets (>60% *f*T_>MIC_) are required. Both low- and higher-dose continuous infusion regimens (Table 4) achieved 100% FTA for both empirical and directed therapy against the P. aeruginosa MIC distribution.

**TABLE 3:** Probability of target attainment for ceftolozane from different ceftolozane-tazobactam dosing regimens^*a*^

PK/PD target	Dose	PTA (%) by MIC (mg/liter):																			
		During the first 24 h										At steady state (48–72 h)									
		0.032	0.064	0.125	0.25	0.5	1	2	4	8	16	0.032	0.064	0.125	0.25	0.5	1	2	4	8	16
40% *f*T_>MIC_	1.5 g q8h	100	100	100	100	100	100	100	100	93	41	100	100	100	100	100	100	100	100	97	59
	1.5 g 4-h EI q8h	100	100	100	100	100	100	100	100	100	52	100	100	100	100	100	100	100	100	100	72
	1.5 g LD + 4.5 g CI	100	100	100	100	100	100	100	100	100	70	100	100	100	100	100	100	100	100	100	68
	3 g q8h	100	100	100	100	100	100	100	100	100	93	100	100	100	100	100	100	100	100	100	97
	3 g 4-h EI q8h	100	100	100	100	100	100	100	100	100	100	100	100	100	100	100	100	100	100	100	100
	3 g LD + 9 g CI	100	100	100	100	100	100	100	100	100	100	100	100	100	100	100	100	100	100	100	100
60% *f*T_>MIC_	1.5 g q8h	100	100	100	100	100	100	100	100	73	3	100	100	100	100	100	100	100	100	78	31
	1.5 g 4-h EI q8h	100	100	100	100	100	100	100	100	82	17	100	100	100	100	100	100	100	100	94	55
	1.5 g LD + 4.5 g CI	100	100	100	100	100	100	100	100	100	68	100	100	100	100	100	100	100	100	100	67
	3 g q8h	100	100	100	100	100	100	100	100	100	73	100	100	100	100	100	100	100	100	100	78
	3 g 4-h EI q8h	100	100	100	100	100	100	100	100	100	82	100	100	100	100	100	100	100	100	100	93
	3 g LD + 9 g CI	100	100	100	100	100	100	100	100	100	100	100	100	100	100	100	100	100	100	100	100
100% *f*T_>MIC_	1.5 g q8h	100	100	100	100	100	100	99	69	5	0	100	100	100	100	100	100	100	81	55	2
	1.5 g 4-h EI q8h	100	100	100	100	100	100	100	69	0	0	100	100	100	100	100	100	100	96	69	10
	1.5 g LD + 4.5 g CI	100	100	100	100	100	100	100	100	98	3	100	100	100	100	100	100	100	100	100	61
	3 g q8h	100	100	100	100	100	100	100	99	69	5	100	100	100	100	100	100	100	100	81	55
	3 g 4-h EI q8h	100	100	100	100	100	100	100	100	72	0	100	100	100	100	100	100	100	100	97	69
	3 g LD + 9 g CI	100	100	100	100	100	100	100	100	100	99	100	100	100	100	100	100	100	100	100	100

a Abbreviations: PK, pharmacokinetic; PD, pharmacodynamic; % *f*T_>MIC_, percentage of time free drug concentration is above the MIC; q8h, every-8-h intermittent infusion (1 h); EI, extended infusion; LD, loading dose; CI, continuous infusion.

**TABLE 4:** Fractional target attainment against P. aeruginosa MIC distribution for steady-state ceftolozane exposure^*a*^

Dose of ceftolozane-tazobactam (2:1 ratio)	PK/PD target	% FTA^*b*^ by urinary creatinine clearance (ml/min/1.73 m^2^) for:							
		Empiric therapy				Directed therapy			
		60	100	140	180	60	100	140	180
1.5 g q8h	40% *f*T_>MIC_	+	+	+	−	+	+	+	+
	60% *f*T_>MIC_	+	+	−	−	+	+	+	+
	100% *f*T_>MIC_	+	−	−	−	+	+	+	−
1.5-g LD + 4.5-g CI	40% *f*T_>MIC_	+	+	+	+	+	+	+	+
	60% *f*T_>MIC_	+	+	+	+	+	+	+	+
	100% *f*T_>MIC_	+	+	+	+	+	+	+	+
3 g q8h	40% *f*T_>MIC_	+	+	+	+	+	+	+	+
	60% *f*T_>MIC_	+	+	+	+	+	+	+	+
	100% *f*T_>MIC_	+	+	−	−	+	+	+	+
3-g LD + 9-g CI	40% *f*T_>MIC_	+	+	+	+	+	+	+	+
	60% *f*T_>MIC_	+	+	+	+	+	+	+	+
	100% *f*T_>MIC_	+	+	+	+	+	+	+	+

a Abbreviations: PK, pharmacokinetic; PD, pharmacodynamic; FTA, fractional target attainment; q8h, every-8-h intermittent infusion (1 h); % *fT*_>MIC_, percentage of time free drug concentration is above the MIC; LD, loading dose over 1 h; CI, continuous infusion over 24 h.

b −, FTA < 85%; +, FTA ≥ 85%.

## DISCUSSION

In this study, we have described the population pharmacokinetics of ceftolozane and tazobactam based on measured unbound concentrations to enable a more robust assessment of the adequacy of recommended dosing regimens for critically ill patients. Given that the free concentration of antibiotics is responsible for the clinical effect, assessment based on direct measurement of unbound concentrations avoids a significant confounding factor when based on total concentration corrected for protein binding. This is because, first, correction for protein binding is often done using a single reported binding ratio uniformly for all patients, disregarding significant between-patient and within-patient variability observed for many drugs (18). Second, there have been discrepancies in the reported binding ratios for ceftolozane in humans (negligible [19, 20], 6.3% [21], and 16 to 21% [17]) and in preclinical studies (5.3% [22] and <5% [23]). Third, binding ratios reported for less sick patients or healthy individuals may not reflect those for critically ill patients because of the high variability in plasma protein concentration and altered binding properties in the critically ill (24, 25). Therefore, the use of unbound pharmacokinetics in this study enables a more reliable prediction of optimal ceftolozane-tazobactam dosing.

The dosing regimen for the approved indication of ceftolozane-tazobactam in cUTI and IAI, a 1.5-g q8h intermittent infusion, achieved high and optimal PTA when considering 40% and 60% *f*T_>MIC_ against MICs as high as 8 mg/liter (Table 3). This is well above the EUCAST *Enterobacterales* (1-mg/liter) and P. aeruginosa (4-mg/liter) clinical breakpoints. For the 40% and 60% *f*T_>MIC_ targets, the 1.5-g q8h regimen also achieves optimal exposure in patients with high creatinine clearance for directed therapy against susceptible P. aeruginosa (Table 4). These results are concordant with previous assessments of the approved dose considering a 32.2% *f*T_>MIC_ target (26, 27). Data from animal model studies show that ∼30 to 40% *f*T_>MIC_ exposure is adequate to achieve a 1- to 2-log kill at 24 h (23, 28), and therefore, a 1.5-g dose is generally appropriate for most patients with susceptible infections. However, in critically ill patients it may be prudent to target a more aggressive exposure of 100% *f*T_>MIC_ (10). Considering this target, the 1.5-g q8h regimen achieved optimal PTA only against MICs of ≤2 mg/liter and optimal FTA only in patients with creatinine clearance of ≤140 ml/min/1.73 m^2^ (for susceptible P. aeruginosa) (Table 4). In other words, this dosage is likely to result in suboptimal exposure in most critically ill patients with augmented renal clearance, even against susceptible P. aeruginosa (13). For empirical coverage against the entire P. aeruginosa MIC distribution, exposures are highly likely to be suboptimal even in patients with average creatinine clearance (e.g., 100 ml/min/1.7 m^2^) (Table 4) if a 100% *f*T_>MIC_ is the desired target.

On the other hand, the 3.0-g q8h intermittent regimen currently licensed for nosocomial pneumonia achieved very high PTA (≥90%) up to an MIC of 8 mg/liter even when considering the aggressive dosing target recommended for the critically ill (100% *f*T_>MIC_) (Table 3). It also achieved the optimal FTA for susceptible pathogens even in patients with augmented renal clearance (Table 4). Therefore, our data strongly suggest that the 3.0-g q8h intermittent infusion regimen is preferable for the treatment of susceptible infections in the critically ill. This is in agreement with Xiao et al., who similarly observed consistently high exposure with a 3.0-g q8h intermittent regimen in their *in silico* simulation study (29). However, for empirical coverage of a suspected P. aeruginosa infection, the 3.0-g q8h regimen achieves a relatively low FTA in patients with severe augmented renal clearance (FTA of 80% for creatinine clearance of 180 ml/min/1.73 m^2^) when targeting 100% *f*T_>MIC_ (Table 4). This may be particularly problematic when using ceftolozane-tazobactam in the management of MDR *Pseudomonas* infections, where the strains may be less susceptible to ceftolozane-tazobactam (30, 31).

To ensure adequate empirical coverage of 100% *f*T_>MIC_ while susceptibility data are pending, the use of continuous-infusion regimens may be highly advantageous. In this study, a 1.5-g loading dose followed by a 4.5-g continuous infusion was adequate to achieve an FTA of ≥85% even in patients with high creatinine clearance (Table 4). For this continuous-infusion regimen, the mean (± standard deviation [SD]) of simulated steady-state unbound ceftolozane concentrations from 48 to 72 h were 11.2 (±3.4) mg/liter and 19 (±5.5) mg/liter for creatinine clearance values of 180 and 100 ml/min/1.73 m^2^, respectively. These values are about three to five times the P. aeruginosa clinical breakpoint (4 mg/liter). Previous studies have shown maximal antibacterial effects for beta-lactam antibiotics when trough concentrations are kept above 4 to 5 times the MIC (32–34). Therefore, a 4.5-g continuous infusion is likely to be highly effective and is supported by clinical case reports demonstrating success against MDR *Pseudomonas* infection susceptible to ceftolozane-tazobactam (35).

Higher doses of a 9.0-g continuous infusion with a 3.0-g initial loading dose result in relatively high average steady-state unbound concentrations of 22.4 (±6.7) and 38 (±11) mg/liter for creatinine clearance values of 180 and 100 ml/min/1.73 m^2^, respectively. Thus, continuous infusion with high-dose regimens is highly likely to consistently achieve high exposure (100% *f*T_>4–5×MIC_) even in patients with augmented renal clearance. This observation is concordant with the recent clinical findings by Pilmis et al. (36) that a 3.0-g (2/1-g) ceftolozane-tazobactam continuous infusion attains 100% *f*T_>4×MIC_ in patients infected with P. aeruginosa up to an MIC of 8 mg/liter. Of note, although there is no clear-cut value for maximum concentration to target, current therapeutic drug monitoring (TDM) practice generally aims to keep a steady-state trough concentration of not more than ten times the MIC as the upper threshold (37). Our results show that continuous infusion with 3.0 g ceftolozane-tazobactam achieves a steady-state unbound concentration of about ten times the MIC clinical breakpoint for P. aeruginosa in patients with average creatinine clearance (about 100 ml/min/1.73 m^2^) or less. For more susceptible isolates with MICs of ≤2 mg/liter, this will be more than twenty times the MIC, clearly above the arbitrary upper threshold common in TDM interventions (37). However, higher-dose continuous infusion may be beneficial in the empirical management of MDR P. aeruginosa infection given that underexposure is likely to trigger resistance *in vivo* during treatment, resulting in reduced susceptibility (30). Such dosing can potentially avoid the treatment failure due to less susceptible strains that is experienced with low-dose intermittent regimen during off-label use (31).

An important limitation in this study is that we have assessed dosing adequacy based on plasma concentrations. While this covers the target site of action for bacteremia, the distribution of ceftolozane in to other sites such as epithelial lining fluid (ELF) in pneumonia could be variable. However, a study (38) recently reported ELF penetration of 97% for ceftolozane, although data in that study were pooled from patients with various levels of renal function to estimate the penetration ratio (the interquartile range of creatinine clearance was 38 to 238 ml/min) and therefore are not likely to reflect a population value extrapolatable to all patients. In a more homogeneous healthy volunteer cohort, a penetration ratio of 0.48 was estimated (39). In either case, given the high PTA up to an MIC of 8 mg/liter (Table 3), adequate exposure will be attainted at the ELF up to the P. aeruginosa breakpoint of 4 mg/liter. Another important limitation of this study is the small sample size, which offers a limited spread of covariates, limiting broad extrapolation of the study findings.

In conclusion, intermittent infusion of 1.5 g ceftolozane-tazobactam q8h achieves adequate unbound plasma exposure against susceptible pathogens. For empirical treatment initiation, intermittent infusion of 3.0 g ceftolozane-tazobactam q8h will be more appropriate and ensures adequate exposure in the lungs given reported penetration ratios of about 0.5 to 1. A loading dose of 1.5 g followed by continuous infusion of 4.5 g is adequate for empirical coverage of a more aggressive dosing target of 100% *f*T_>MIC_, including in patients with augmented renal clearance.

## MATERIALS AND METHODS

### Study design and setting.

This prospective observational pharmacokinetic study was conducted at a quaternary referral intensive care unit (ICU) of the Royal Brisbane and Women’s Hospital (RBWH), Australia. The human research ethics committees of RBWH (HREC/16/QRBW/211) and the University of Queensland (no. 2016001368) granted ethical clearance.

### Patients.

ICU patients, aged ≥18 years, were enrolled if diagnosed with a systemic infection known or suspected to be caused by a bacterium susceptible to ceftolozane-tazobactam. Patients were excluded if they had renal dysfunction that necessitated the use of renal replacement therapy, had a known or suspected allergy to cephalosporins, had received piperacillin-tazobactam in the preceding 7 days, or were pregnant. Informed consent was obtained from each patient or their legally authorized representative.

### Ceftolozane-tazobactam administration.

At the discretion of the treating physician, the study participants received either 1.5 g or 3.0 g ceftolozane-tazobactam (2:1 ratio) administered every 8 h via intravenous infusion over 1 h. The attending clinicians determined the duration of therapy based on the patients’ clinical scenario.

### Sample collection.

Blood samples (3 ml each) were collected in heparinized Vacutainers from an established arterial line. The sampling times were as follows: first sample just prior to administration of the dose; second and third samples at 15 and 45 min, respectively, after commencement of drug infusion; fourth sample at the end of line flushing (15 to 20 min) following the 1-h drug infusion; samples at 2, 3, 4, 5, 6, and 7 h after the start of infusion; and a final sample just before the second dose. The actual time of collection for individual samples was recorded and used for analysis. Blood samples were spun (3,000 rpm for 10 min) immediately after collection to separate plasma, an aliquot of which was stored in a –80°C freezer until assayed by a validated chromatographic method.

### Clinical data.

An electronic case report form developed in the REDCap web platform was used to collect clinical data, including the following: patient demographics; physical examination, including vital signs; ICU and hospital admission and discharge dates and times; Acute Physiology and Chronic Health Evaluation II (APACHE II) score; Sequential Organ Failure Assessment (SOFA) score at ICU admission; presence of shock on days of sampling; presence of mechanical ventilation; renal function markers (serum creatinine concentration and urinary creatinine clearance); liver laboratory test results (alanine aminotransferase, aspartate aminotransferase, alkaline phosphatase, gamma glutamyl transferase, international normalized ratio, and bilirubin); medication list on days of sampling; antibiotic data, including type, dose, dosing interval, duration of infusion, and other antibiotics administered on day of sampling; and infection data (organisms isolated and sample type, MIC if available).

### Ceftolozane-tazobactam assay.

Unbound concentrations of ceftolozane and tazobactam in plasma were measured by an ultra-high-performance liquid chromatography–tandem mass spectrometry (UHPLC-MS/MS) method on a Shimadzu Nexera2 UHPLC system coupled to a Shimadzu 8050 triple-quadrupole mass spectrometer (Kyoto, Japan). The unbound fraction of plasma was isolated by ultracentrifugation using Centrifree devices (Millipore, Tullagreen, Ireland). The sample (10 μl) was spiked with phosphate-buffered saline (pH 7.4), an internal standard (sulbactam and l-cefazolin), and acetonitrile. The stationary phase was a C_18_ Ultra IBD column (100 by 2.1 mm, 3 μm) (Restek, USA) operated at room temperature. Mobile phase A was 0.1% (vol/vol) formic acid in 10 mM ammonium formate, and mobile phase B was 100% acetonitrile with 0.1% (vol/vol) formic acid. The mobile phase was delivered with gradient from 15% to 50% B at a flow rate of 0.3 ml/min for a 5-min run time and produced a back pressure of approximately 2,800 lb/in^2^. Ceftolozane was monitored by positive-mode electrospray at MRMs of 667.00→199.15. Labeled cefazolin was monitored in positive mode at 457.85→326.05. Tazobactam and sulbactam were monitored by negative-mode electrospray at MRMs 299.20→138.00 and 232.20→140.00, respectively. The calibration range for ceftolozane was 1 to 100 mg/liter, and that for tazobactam was 0.5 to 100 mg/liter. For ceftolozane at total concentrations of 160, 20, and 3 mg/liter, the precision of the unbound analysis was 6.3, 6.2, and 8.2% with unbound fractions of 90%, 99%, and 101%. For tazobactam at total concentrations of 80, 10, and 1.5 mg/liter, the precision of unbound analysis was 6.2, 7.5, and 8.1% with unbound fractions of 89, 91, and 92%. The assay method was validated using the FDA criteria for bioanalysis (40).

### Population PK modeling.

A population pharmacokinetic (PK) model was developed in R using Pmetrics version 1.5.2. Unbound ceftolozane and tazobactam concentration-time data were modelled using nonparametric adaptive grid (NPAG) analysis in Pmetrics. Initially, one- and two-compartment structural base models were tested considering first-order elimination from the central compartment and intercompartmental distribution. With each structural base model, either a multiplicative or additive error model was tested. The additive error mode was given by the equation Error = (SD2 + λ2)0.5, and the multiplicative mode was given by the equation Error = SD · γ, where SD represents the standard deviation of observations and λ and γ represent process noise. In addition, assay error was modelled as a linear function of observations (obs) as Error = *C*0 + *C*1 · obs, where the coefficients *C*0 and *C*1 were optimized interactively.

Covariate models were tested following the standard forward-addition and backward-deletion approach. Initially, covariates were selected based on biological plausibility as well as a preliminary regression analysis of each plausible covariate against primary model parameters using built-in tools within Pmetrics. Covariates selected for investigation include serum creatinine, urinary creatinine clearance, body weight, body mass index, albumin concentration, Acute Physiology and Chronic Health Evaluation II (APACHE II) score, and Sequential Organ Failure Assessment (SOFA) score. Model evaluation and selection were based on assessment of diagnostic plots and statistics. Diagnostic plots included observed versus population or individual predicted concentrations and normalized prediction distribution errors (NPDE) versus time or observation plots. Statistics included regression coefficient of observed versus predicted concentrations, bias [defined as the mean weighted error of predicted minus observed concentrations, i.e., Σ(predicted − observed/standard deviation)/*N*], imprecision {defined as the bias-adjusted, mean weighted squared error of predicted minus observed concentration, i.e., Σ[(predicted − observed)^2^/(standard deviation)^2^]/*N* − Σ(predicted-observed)/standard deviations/*N*, where *N* is the number of observations/predictions), and objective functions, including log-likelihood ratio (LLR) test for the nested models, Akaike information criterion (AIC), and Bayesian information criterion (BIC). The LLR chi-square test was used for statistical comparison of nested models (a *P* value of <0.5 was considered significant).

### Dosing simulations.

Using the final covariate model, Monte Carlo dosing simulations (*n* = 1,000) were performed to determine the probability of target attainment (PTA) during the first 24 h and at steady state from 48 to 72 h after commencement of treatment. Simulated dosing regimens of ceftolozane-tazobactam (2:1 ratio) included a 1.5-g intermittent infusion (over 1 h) every 8 h (q8h), a 1.5-g extended infusion (over 4 h) q8h, a 1.5-g loading dose over 1 h plus a 4.5-g continuous infusion over 24 h, a 3-g intermittent infusion (over 1 h) q8h, a 3-g extended infusion (over 4 h) q8h, and a 3-g loading dose over 1 h plus a 9-g continuous infusion over 24 h.

The primary pharmacokinetic (PK)/pharmacodynamic (PD) dosing target used for determination of PTA for ceftolozane was 40% *f*T_>MIC_. This is based on preclinical studies that showed that a 32.2% *f*T_>MIC_ exposure achieves a 1-log kill (23) and that a 40% to 50% % *f*T_>MIC_ is likely to achieve a 1- to 2-log kill (28). In addition, we determined the PTA for a higher exposure of 60% *f*T_>MIC_, which is generally considered optimal for cephalosporins (41), and a more aggressive exposure of 100% *f*T_>MIC_, which is advocated as a prudent target for severely ill patient populations (10). For tazobactam, we used a 20% *f*T_>1mg/liter_ (20% of the time above the minimum effective concentration of 1 mg/liter) as a target for assessment of dosing adequacy as previously suggested based on data from preclinical studies (26, 27, 42).

The cumulative fractional response or fractional target attainment (FTA) for ceftolozane was estimated for the Pseudomonas aeruginosa EUCAST MIC distribution for both empirical and directed therapy using the equation $\mathrm{FTA}=\sum_{i=0.125}^{n}PTA_{i}\times F_{i}$, where *i* is the MIC category ranging from 0.125 to *n*, *n* is 64 mg/liter for empirical therapy and the EUCAST clinical breakpoint of 4 mg/liter for directed therapy, *PTA_i_* is the PTA for MIC category *i*, and *F_i_* is the fraction of the bacterial population at each MIC category.
